# Mental rotation of sequentially presented 3D figures: sex and sex hormones related differences in behavioural and ERP measures

**DOI:** 10.1038/s41598-019-55433-y

**Published:** 2019-12-11

**Authors:** Ramune Griksiene, Aurina Arnatkeviciute, Rasa Monciunskaite, Thomas Koenig, Osvaldas Ruksenas

**Affiliations:** 1 Department of Neurobiology and Biophysics, Vilnius University, Vilnius, Lithuania; 2 Translational Research Center, University Hospital of Psychiatry, Bern, Switzerland

**Keywords:** Cognitive control, Human behaviour

## Abstract

Mental rotation of 3D objects demonstrates one of the largest sex differences. We investigated sex and sex hormones-related differences in behaviour and event related potentials (ERP) using a modified Shepard and Metzler task composed of sequentially presented 3D figures in 29 men and 32 women. We demonstrated a significant increase in response time and decrease in both accuracy and positivity of the parietal ERP with increasing angular disparity between the figures. Higher angular disparity evoked an increase of global field power (GFP) from 270 to 460 ms and different activation topographies from 470 to 583 ms with lower parietal, but higher left frontal positivity. Flatter slopes in higher angular disparity condition suggest distinct strategies being implemented depending on the difficulty of the rotation. Men performed the task more accurately than women. Performance accuracy in women tended to be negatively related to estradiol while the response time tended to increase with increasing progesterone. There were no associations with testosterone. Women demonstrated higher GFP and an increased positivity over the parietal scalp area, while men showed higher activation in the left frontal cortex. Together these findings indicate dynamic angular disparity- and sex-related differences in brain activity during mental rotation of 3D figures.

## Introduction

Mental rotation tasks, in which an object is rotated in the three-dimensional (3D) space, have been shown to produce consistent sex differences in favour of males^1–6^. Males surpass females at the behavioural level demonstrating higher accuracy and occasional shorter response times^2,7–13^. Functional magnetic resonance imaging (fMRI) studies reveal brain activity differences driven by sex and/or sex steroids^5,14–18^. However, the consensus regarding sex differences in brain activation during mental rotation still has not been reached, as many studies demonstrate higher levels of activation in the parietal region in males^5,14,15,17–19^, while others observe higher activations in the frontal lobe in females^14,15,18^, or do not find any significant differences at all^17,20^.

Next to the general sex differences, sex hormones can influence spatial cognition. The fluctuation of sex steroids during women menstrual cycle has been reported to affect mental rotation performance: increase of estradiol level was related to decrease in accuracy^21,22^ and higher progesterone level - to slower responses^10,23^. Inversely, the performance of both men^24^ and women^22,25^ were positively related with the presence of androgens. Studies showed a female-like neural activation pattern in mental rotation task among subjects with a complete androgen insensitivity syndrome (karyotyoe in males and phenotype in females)^17^ and male-to-female transsexuals after a cross-sex hormone treatment^26^. These results indicate that sex differences in regional brain function during mental rotation are influenced by sex steroids. In contrast, there are mental rotation studies failing to provide evidence of association between gonadal hormones and sex differences in task performance^4,27^.

The attempts to answer when, where and what kind of differences manifest between men and women during this sex-specific task may help to develop a much better understanding of their mechanisms and the effects of sex steroids on human cognition.

Building on findings from behavioural and neurophysiological studies of mental rotation tasks, the differences in task performance between males and females have been proposed to result from sex-related preference of different strategies. To systematically probe the strategies that may have been used to solve the task, experimenters have employed a series of methods, such as the variation in the complexity of the stimuli^28^, the self-report questionnaires about the strategies that participants used^5,29^, different instructions^8^, the evaluation the slope of response time^8,30,31^, or the evaluation of brain activity during mental rotation^5,16,18^. These analyses suggested that males were biased towards a global evaluation and whole-object rotation strategy, while females more often employed rotation-independent piecemeal strategies, such as counting blocs or noting the relative orientations of the object parts^8,15,16^. The evaluation of sex differences in strategies used to perform mental rotation task may benefit from better understanding of the sub-processes of mental rotation. Mental rotation recruits several sub-processes on a millisecond time scale, including perception, assessment of the complex stimulus aspects (categorization), rotation, and decision^32,33^. It is possible that involvement and/or timing of these sub-processes differs between men and women, thus causing the overall difference in performance. Time-resolved fMRI analyses have been used to evaluate the activation of cortical brain networks in relation to specific functions during the mental rotation process^34,35^. Visual system components (the lateral occipital complex, dorsal extrastriate visual areas) have shown increased activity during visual perception^34^; frontal areas (such as the supplementary motor area, the bilateral premotor cortex) and the parietal cortex may be involved to the computation of visuo-spatial transformation^34,35^; whereas the activation of the primary motor cortex was related to a button press^35^. Nevertheless the rapid temporal succession of such sub-processes cannot be precisely discriminated using relatively low temporal resolution neuroimaging methods^36,37^. Whereas the high temporal resolution of the electroencephalography (EEG) allows for a decomposition of the cognitive process into a sequence of processing stages using information from the event related potentials (ERPs)^38^.

Many studies have used ERP paradigms to identify neurophysiological correlates of mental rotation (e.g.^32,33,39–41^) demonstrating similar ERP waveform patterns across studies, despite numerous methodological differences (various stimuli, presented or not presented standards, with or without time limits, etc.). Based on the early work presented by Desrocher *et al*.^32^ and some later studies, ERP waveform components may be interpreted as showing several aspects of the mental rotation process, namely: (i) processing of a sensory information and evaluation of simple aspects of a stimulus (occurs within 100–300 ms after stimulus onset, possibly involves an activation of occipital, parietal and frontal areas)^32,40,42,43^; (ii) assessment of a complex stimulus aspects and selection of a strategy (300–400 ms, frontal areas)^32,40,44^; (iii) rotation (400–800 ms, centro-parietal areas)^32,40,45^, (iv) decision and motor response (700 ms – until response, centro-parietal and frontal areas)^32,42^). However, the most of the mental rotation ERP studies concentrated on a particular time period and/or scalp area^33,41,46–48^. These types of studies reported a late, slow, and consistent decrease in positivity (or increase in negativity) with increasing angular disparity in the time window from approximately 350 ms to approximately 800 ms, over the centro-parietal scalp area that was considered a direct electrophysiological index of the rotation sub-process of the mental rotation (e.g.^32,40,41,45,46,48^) and named rotation related negativity (RRN)^48^.

Even though there is an extensive body of literature investigating general neurophysiological correlates of mental rotation, only very few ERP studies were conducted to investigate sex differences and the results are inconsistent. Desrocher *et al*.^32^ found no clear sex-related behavioural differences during the rotation of letters and abstract two-dimensional (2D) figures, but demonstrated higher P3 (at Cz, in approximately 400–700 ms time window) amplitude in women. At the same time, no sex differences in performance and P3 (250–600 ms) amplitude were found in a complex mental rotation task (Spatial Folding and Cutting subtest of the Stanford-Binet IQ test), however, men had shorter P3 and longer P1 (40–120 ms) latencies as well as lower N1 (120–220 ms) amplitudes compared to women^33^. Gootjes *et al*.^40^ demonstrated approximately 100 ms longer response times in women using a lowercase letter rotation task. This response time delay corresponded to an approximately 100 ms latency increase of the late (400–800 ms) mental rotation-related ERP component in women compared to men, suggesting that women processed mental rotation later. Moreover, greater amplitude at the midline electrodes for women at time interval from 130 to 270 ms suggests that sex differences in relatively early processing stages are probably related to perception and identification. However, Beste *et al*.^39^ reported that sex had no effect on the mental rotation of characters, neither with respect to performance nor to ERPs. Arguing that characters are not the most reliable stimuli to study sex differences in mental rotation, Pellkofer *et al*.^49^ used simultaneously presented polygons and demonstrated sex differences in both performance and sex-specific functional brain asymmetry, with bilateral brain activity in parietal areas in men and left-lateralized brain activity in women.

To the best of our knowledge, only two studies compared ERP-related brain activity between men and women during mental rotation of 3D objects^50,51^. This task has been shown to demonstrate the most consistent sex differences in performance. Wegesin^50^ analysed a very late (1800–3700 ms after stimuli onset) parietal ERP component and showed a more negative wave in men than in women. The study presented by Yu *et al*.^51^ did not demonstrate sex differences in parietal ERPs, but a more negative right frontal ERP amplitude in women in the time interval from 400 to 700 ms. Importantly, these sex differences appeared earlier than the mental rotation-related effect (600–1000 ms, frontal and parietal areas) leading to an interpretation that sex differences occurred in cognitive processing stages related to perception, stimuli identification, and strategy selection.

The lack of studies combining 3D stimuli with ERPs may be related to the incompatibility between the most popular 3D mental rotation tasks and ERP measurements. For example, the task based on the Vandenberg and Kuse paradigm^52^, which is known to demonstrate the largest behavioural sex differences in 3D mental rotation^53^, is incompatible with ERPs due to the absence of clear time restrictions and extensive ocular movements while the target is compared with four comparison figures. In the above mentioned 3D mental rotation studies^50,51^, the classical Shepard and Metzler^54^ paradigm has been implemented. In the Shepard and Metzler paradigm, subjects are presented with a pair of asymmetrical figures with a task to decide whether the two figures are the same or different. The comparison of two simultaneously presented figures evokes horizontal eye movements and overt attention shifts. Moreover, it has been demonstrated that the increase in angular disparity during mental rotation is associated with changes in saccadic activity^46,55^. Saccades and shifts in attention between visual hemifields have been shown to produce systematic frontal and parietal activation^56,57^ that can interfere with the mental rotation-related activation. In addition, EEG signals are contaminated with the eye dipole artefact which is evoked by saccades in each trial and is systematically task-dependent. No artefact correction technique is refined enough to remove eye movement artefacts of such extent^38^. For this reason, the EEG studies should avoid using, where possible, tasks that require constant eye movement in each trial.

**Figure 1:**
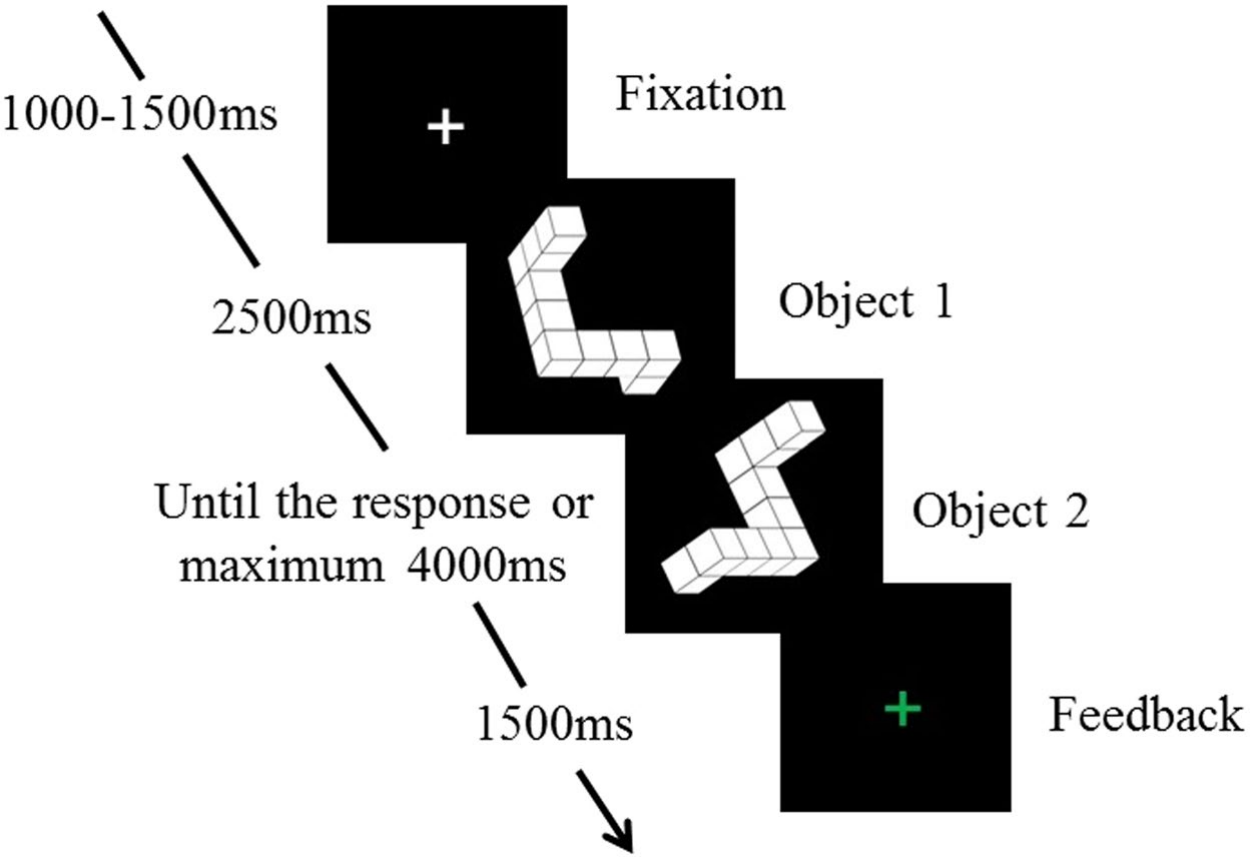
A schematic time course of a single trial in the mental rotation task. Each trial began with the presentation of a fixation cross for 1000–1500 ms. Object 1 appeared immediately thereafter and was present for 2500 ms followed by Object 2 which remained on the screen until the response or for 4000 ms in case of no response. Participants were instructed to respond with the dominant hand: a green button for identical and a red for mirror figures. Reprinted from^92^ with permission from Elsevier.

Considering these issues, we used a modified 3D mental rotation paradigm (Fig. 1) to assess behavioural and ERP differences between men and women. We modified the Shepard and Metzler task by presenting two 3D figures sequentially instead of in parallel. Such a modification avoids saccades and attention shifts during the trial, as only a single stimulus is presented at a time. This task is somewhat similar to the successive presentation task used by Cohen and Kubovy using 2D shapes (polygons and matrices)^30^. Increasing rotation rate (i.e. a rise in response time with increasing angular disparity) is a marker of the mental rotation proper^24,54^. Comparing the conditions with simultaneous and successive presentation of the stimulus, Cohen and Kubovy^30^ observed that a response time increased with increasing angular disparity in both cases, with a slightly less expressed effect in the successive presentation paradigm. The presence of such increase using successive presentation confirms that this paradigm evokes the same process of mental rotation, as does the simultaneous presentation. Nevertheless, as our task markedly differed from the classical tasks used in most mental rotation studies, we first aimed to confirm the appropriateness of the sequential presentation task for the behavioural and ERP study of mental rotation by evaluating the effect of angular disparity. Based on many previous mental rotation studies, we expected higher angular disparity conditions to be associated with a lower accuracy, a longer response time and a lower amplitude of the late, rotation-related, ERP component regardless of sex.

Next, we investigated the sex effect on the behavioural and electrophysiological measures (ERPs) during 3D mental rotation. As the main behavioural data, we evaluated the performance accuracy and response time. We hypothesized that women would perform the task less accurately. For EEG data, alongside classical ERP waveform analyses, we investigated ERPs across the entire scalp and the whole epoch. We measured Global Field Power (GFP), which represents the quantitative evaluation of sources activity^58^, and performed topographic analysis of variance (TANOVA), which enables the evaluation of topographic effects and represents qualitative neural activity differences^59^. Concerning the literature linking sex differences in mental rotation with the implementation of different strategies, (e.g.^8,15,16^), we expected to observe strategy-related differences. In our study, we instructed participants to rotate the figures in their mind seeking to encourage the use of a rotation as a strategy to perform the task. We then evaluated the behavioural and ERP data to discover retrospectively the strategy used. We calculated slopes of the response time. Steep slopes are assumed to reflect a rotation strategy, whereas shallow slopes possibly reflect other (e.g. piecemeal) strategies^8,30,31^. For the ERP data, we predicted to see a reflection of different strategies from the time course of ERP and from the TANOVA. We anticipated to observe sex differences in brain activation during the pre-rotational time period potentially associated with strategy selection (approximately 300–400 ms)^32,44^ and the rotation-related time window (approximately 400–800 ms)^32,40,41,45,46,48^. We also expected to see significant differences in activation topographies which may suggest that at least partially different sources have been involved^60–62^.

Finally, as a number of behavioural and fMRI studies suggested that the levels of estradiol, progesterone and testosterone have significant influence on the performance and associated brain activity during mental rotation^5,21,23^, we aimed to assess the relationship between sex steroids and the ERPs during the mental rotation of 3D objects. In regards to the sex steroids, we expected testosterone to exert a positive effect on task performance. The effect of estradiol and progesterone was expected to be negative, with a possible decrease of accuracy with increasing estradiol, and rising response time with increasing progesterone. Given the lack of previous data on the relationship between hormones and ERPs parameters, we treated this part of the analysis as exploratory.

## Results

### Hormone levels

The data of salivary 17β-estradiol, progesterone and testosterone levels during follicular and luteal phases in women and the level of salivary testosterone in men are provided in Table 1. The mean 17β-estradiol and progesterone concentrations in follicular and luteal phases were close to the values expected from the literature (e.g.^63,64^). However, in line with previous studies (e.g.^65–67^), individual hormonal values for some women did not correspond to the expected menstrual cycle phase. Based on the recommendations for salivary progesterone ELISA assays (IBL International) and the literature^68^, we expected that the level of progesterone in the mid-luteal phase will vary within a range from 100 to 450 pg/ml. There were seven women with a lower than expected level of progesterone in the mid-luteal phase (Table 1). We thus subsequently decided not to divide women into follicular and luteal phase groups, but rather to evaluate the relationships between the concentrations of sex hormones and behavioural data and ERP parameters.

**Table 1:** Hormone levels in men and women in both follicular and luteal phases.

	Men	Women		
		Foll phase	Lut phase	t-test
Progesterone, pg/ml	na	58.4 ± 53.3 min 15.4, max 86.5	132.4 ± 79.1 min 33.2, max 322.0	t = −2.72, p = 0.01, d = 1.10
17β-estradiol, pg/ml	na	3.66 ± 1.44 min 2.02, max 7.26	4.04 ± 1.13 min 2.47, max 7.51	*ns*
Testosterone, pg/ml	174.2 ± 81.9 min 47.8, max 345.5	42.9 ± 12.1 min 32.9, max 67.6	43.6 ± 24.4 min 15.4, max 88.1	Men *vs* Women t ≥ 7.72, p < 0.001, d ≥ 2.16Foll *vs* Lut *ns*

Note. Mean and SD, minimum (min) and maximum (max) values are shown in the table; na – not assessed; ns – not significant; Foll – follicular phase, Lut – luteal phase.

### Behavioural results

For behavioural data of the mental rotation task (Fig. 1), we evaluated the performance accuracy, response time of correct trials, and calculated slopes of the response time. In the whole analyses (behavioural and ERP data), we included only data from identical trials, i.e. trials where two figures (asymmetrical assembles of ten cubes) in pair were identical (not a mirror image of each other), despite their angular disparity.

A Mixed design analysis of covariance (Mixed design ANCOVA) was used to evaluate the effect of angular disparity (25°, 50°, 75°, 100°, 125°, and 150°) and sex (men *vs* women). Mauchly’s test indicated that the sphericity assumption has been violated for angular disparity for ACC data χ^2^ (14) = 34.3, p = 0.002, and for the RT data, χ^2^ (14) = 40.7, p < 0.001. Therefore, degrees of freedom were corrected using Greenhouse-Geisser estimates of sphericity for ACC (ε = 0.803) and RT (ε = 0.746).

The results showed a significant main effect of angular disparity on ACC (F(4.02, 245.0) = 24.56, p < 0.0001, η^2^ = 0.29) and RT (F(3.73, 227.7) = 35.2, p < 0.0001, η^2^ = 0.37). Accuracy decreased and response time raised with increasing angular disparity between figures in a pair.

**Figure 2:**
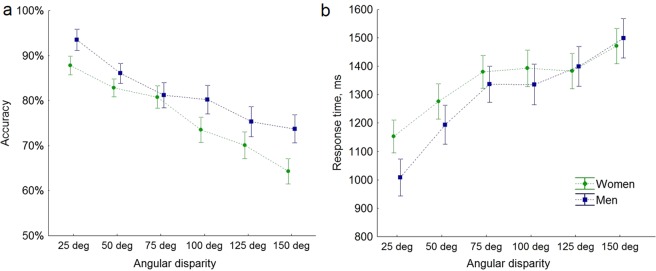
Accuracy (%) (**a**) and response time (ms) (**b**) as a function of the angular disparity between the two figures for men and women. Vertical bars denote standard errors of the mean (SE).

The effect of sex was significant for ACC (F(1, 61) = 4.50, p = 0.038, η^2^ = 0.29), but not RT (F(1, 61) = 0.87, p = 0.35, η^2^ = 0.01). On average, men were significantly more accurate (81.4 ± 10.5 SD%) than women (76.1 ± 10.3 SD%), while the mean response time was very similar (men 1260 ± 276 SD ms, women 1297 ± 357 SD ms) (Fig. 2).

No significant interaction between main factors was found for ACC (F(4.0, 245.0) = 1.24, p = 0.294, η^2^ = 0.02) and RT (F(3.7, 227.7) = 1.31, p = 0.270, η^2^ = 0.02). Also, the covariates such as age, the duration of education, spatial, and math abilities did not have a significant effect on ACC (all F < 3.2, all p ≥ 0.08) or RT (all F < 1.45, all p > 0.23).

T-tests did not show sex differences for the slope (men 3.50 ± SD 2.56 ms/deg, women 3.19 ± SD 1.99 ms/deg, p = 0.60, d = 0.14) nor for the intercept (men intercept 953 ± SD 257 ms, women 988 ± SD 337 ms, p = 0.65, d = 0.12) of the response time.

As shown in Fig. 2b, the slope started to flatten above the 75 deg of angular disparity for both men and women. Therefore, we computed the slope values for lower (25, 50 and 75 deg) and higher (100, 125 and 150 deg) angular disparities and applied a 2 (low *vs* high disparity) × 2 (male *vs* female) ANOVA to evaluate an effect of these factors. The effect of angular disparity was significant (F(1, 118) = 14.45 p < 0.001, η^2^ = 0.12), i.e. the slope was steeper in lower (5.84 ± SD 3.54 ms/deg) than in higher (2.42 ± 4.33 ms/deg) angular disparity condition. Neither the effect of sex (p > 0.18) nor interaction (p > 0.98) between these two factors was significant.

### Event related potentials

The analysis of the ERP data was limited to the identical trials followed by correct response (77.27% of all identical trials responses were correct, 22.57% - incorrect, and in 0.16% of trials no response was provided).

The main analyses applied on ERPs (GFP and TANOVA) revealed significant effects of both main factors (angular disparity and sex) but no significant interaction between them. Therefore, the ERP results are presented separately for each factor. In the first part, we describe the effect of the angular disparity regardless of sex, while in the second part the effect of sex is evaluated independent of the angular disparity.

#### The effect of angular disparity

A pilot inspection of the ERPs revealed that the data for 25° angular disparity trials did not follow the common pattern of other angular disparity results (for details see Appendix A, where the examples of six angular disparity conditions and averaged ERP waveforms from the parietal scalp area electrodes in all six angular disparity conditions are presented). Accordingly, we removed 25° angular disparity trials from the part of the analysis where the effect of angular disparity was evaluated. Then, to simplify data categorization and ensure an adequate signal-to-noise ratio in the ERPs, we separated trials into low (trials of 50° and 75°) and high (trials of 125° and 150°) angular disparity conditions. The trials with 100° angular disparity appeared in the middle and were therefore removed from this part of the analysis. Consequently, for the evaluation of the angular disparity effect, individual averages of ERPs were computed for low and high angular disparity conditions. The number of valid trials differed significantly between low and high angular disparity conditions (F(1, 59) = 46.8, p < 0.001, η^2^ = 0.44). Due to the lower accuracy at high angular disparity, fewer correct trials were available (mean_high_ = 12.20 ± 1.97 valid epochs per subject, min 10, max 19 compared to mean_low_ = 13.95 ± 1.85, min 11, max 18). Group (men *vs* women) had no significant effect on the number of valid epochs per condition (p = 0.57) and there was no significant interaction between group and condition (p = 0.81).

**Figure 3:**
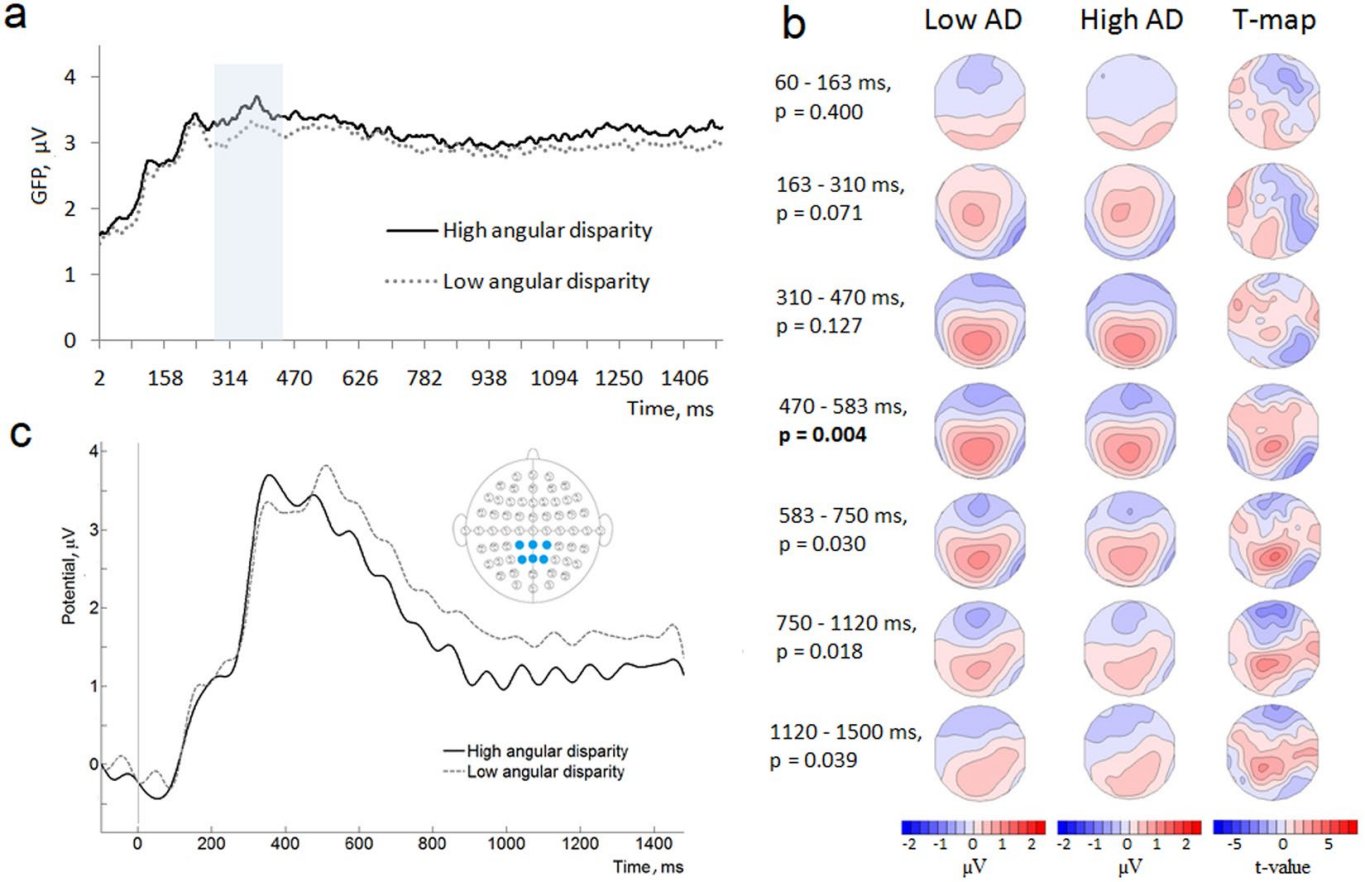
The comparison of the ERPs in low *vs* high angular disparity (AD) conditions in all subjects: (**a**) GFP values (vertical axis) as a function of time (horizontal axis) for high and low angular disparities. Blue background denotes the time window (from 270 to 460 ms) where the effect was statistically significant (p < 0.05, randomization statistics); (**b**) ERP scalp distributions and t-maps contrasting low minus high angular disparities in 7 time periods after the stimulus onset; p-values (randomization statistics) indicating significant differences are marked in bold. Blue map areas indicate negative, while red indicate positive values; (**c**) Averaged ERP waveforms from the parietal scalp area electrodes (CP1, CPz, CP2, P1, Pz, P2) in low and high angular disparity conditions.

First, we evaluated the difference in GFP between low and high angular disparity conditions followed by the TANOVA to determine the qualitative differences in topology. High angular disparity condition demonstrated higher GFP in the time window from 270 to 460 ms (p < 0.05, randomization statistics) (Fig. 3a). TANOVA showed differences between the angular disparity conditions in the time window from 470 ms until the end of the epoch (1500 ms) (Fig. 3b). However, only in a period from 470 ms to 583 ms the differences were significant, i.e. survived the Bonferroni correction (p < 0.007). The spatial distribution of the effect is displayed with t-maps (Fig. 3b). In the time window from 470 to 583 ms, t-map contrasting low minus high angular disparities showed bilateral centro-parietal positivity with a maximal t-value at the CPz electrode (t = 4.09) and a right parietal negativity with a minimal t-value at the P8 electrode (t = −3.39).

In order to compare our results to the previous mental rotation ERP studies^41,43,48,69^, we averaged ERP waveforms over the parietal scalp area electrodes (CP1, CPz, CP2, P1, Pz, P2) in low and high angular disparity conditions (Fig. 3c). In line with previous studies, a decrease in positivity for the high angular disparity condition was found. As the statistical significance of the effect was already proven by TANOVA, we did not perform additional statistical tests for the amplitudes.

**Figure 4:**
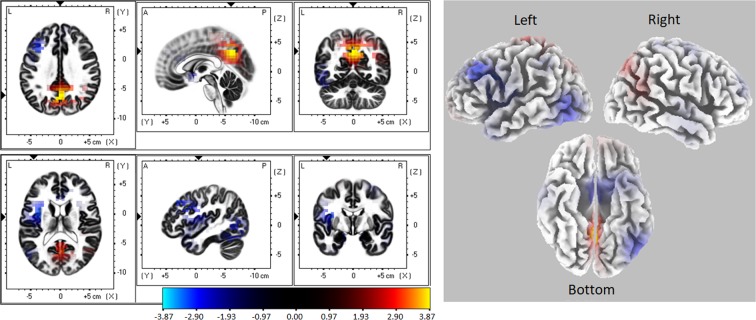
Differences in estimated source activity between low and high angular disparity conditions (low minus high). The differences were calculated in the time window from 470 to 583 ms after stimulus onset. Areas marked red and yellow indicate higher activation in low angular disparity condition; areas marked blue indicate higher activation in high angular disparity condition.

Source activity was compared only in the time window where TANOVA showed a significant condition effect (from 470 to 583 ms). The sLORETA analysis revealed significant differences in source activation between low and high angular disparity conditions (Fig. 4 and Table 2). Low angular disparity condition showed increased source activation in the precuneus (the postero-medial portion of the parietal lobe) and cingulated gyrus while the high angular disparity condition was characterized by some lateralized activity in the left hemisphere (insula, frontal, temporal and occipital gyrus) (Fig. 4 and Table 2).

**Table 2:** Regions showing significant angular disparity related differences (low minus high angular disparity) in the time window from 470 to 583 ms after stimulus onset.

X(MNI)	Y(MNI)	Z(MNI)	Voxel t-value	BA	Lobe	Structure
0	−60	35	3.87	7	Parietal	Precuneus
−5	−60	30	3.7	31	Limbic	Cingulate gyrus
−35	5	20	−3.24	13	Sub-Lobar	Insula
−35	20	35	−3.07	9	Frontal	Middle Frontal Gyrus
−55	−65	0	−2.59	37	Temporal	Middle Temporal Gyrus
−55	−70	5	−2.56	19	Occipital	Middle Occipital Gyrus

Positive t-values indicate higher activation in low angular disparity condition; negative t-values indicate higher activation in high angular disparity condition.

All regions were thresholded at p < 0.05 after randomization procedure.

BA Brodmann’s area, MNI Montreal Neurological Institute.

#### Sex effect

Effects of sex were tested by comparing GFP and topographies between men and women. Both GFP and ERP scalp distributions evaluated using TANOVA did not show any significant differences during the baseline period, i.e. the time period from −200 ms to 0 ms with respect to the Object 1 before the baseline correction: GFP_men_ = 2.17 ± 0.20 SE µV, GFP_women_ = 2.67 ± 0.18 SE µV, p = 0.21; TANOVA (p = 0.33).

The effect of sex on GFP and topographies was then evaluated in each of the seven microstate-defined time periods in a time interval from 0 to 1500 ms after stimulus (Object 2) onset using data averaged over all (six) angular disparity conditions.

**Figure 5:**
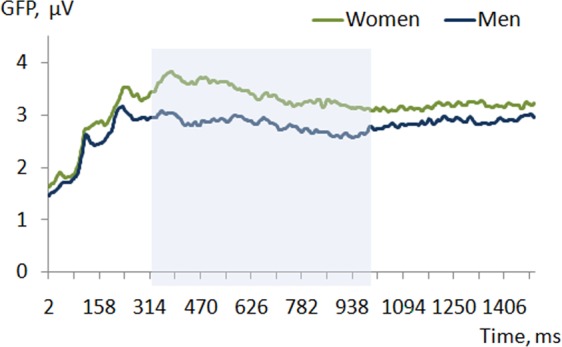
The comparison of GFP between women and men GFP values (vertical axis) as a function of time (horizontal axis) averaged across all angular disparity conditions. Blue background denotes the time window (from 320 to 990 ms) where p < 0.05 (randomization statistics).

Women demonstrated higher GFP during the whole epoch with a significant increase in the time window from 320 to 990 ms (Fig. 5). We also observed qualitatively different GFP patterns over time with two characteristic peaks in women (230 ms and 380 ms), while men showed a relatively steady decrease from around 230 ms after the stimulus onset (Fig. 5).

**Figure 6:**
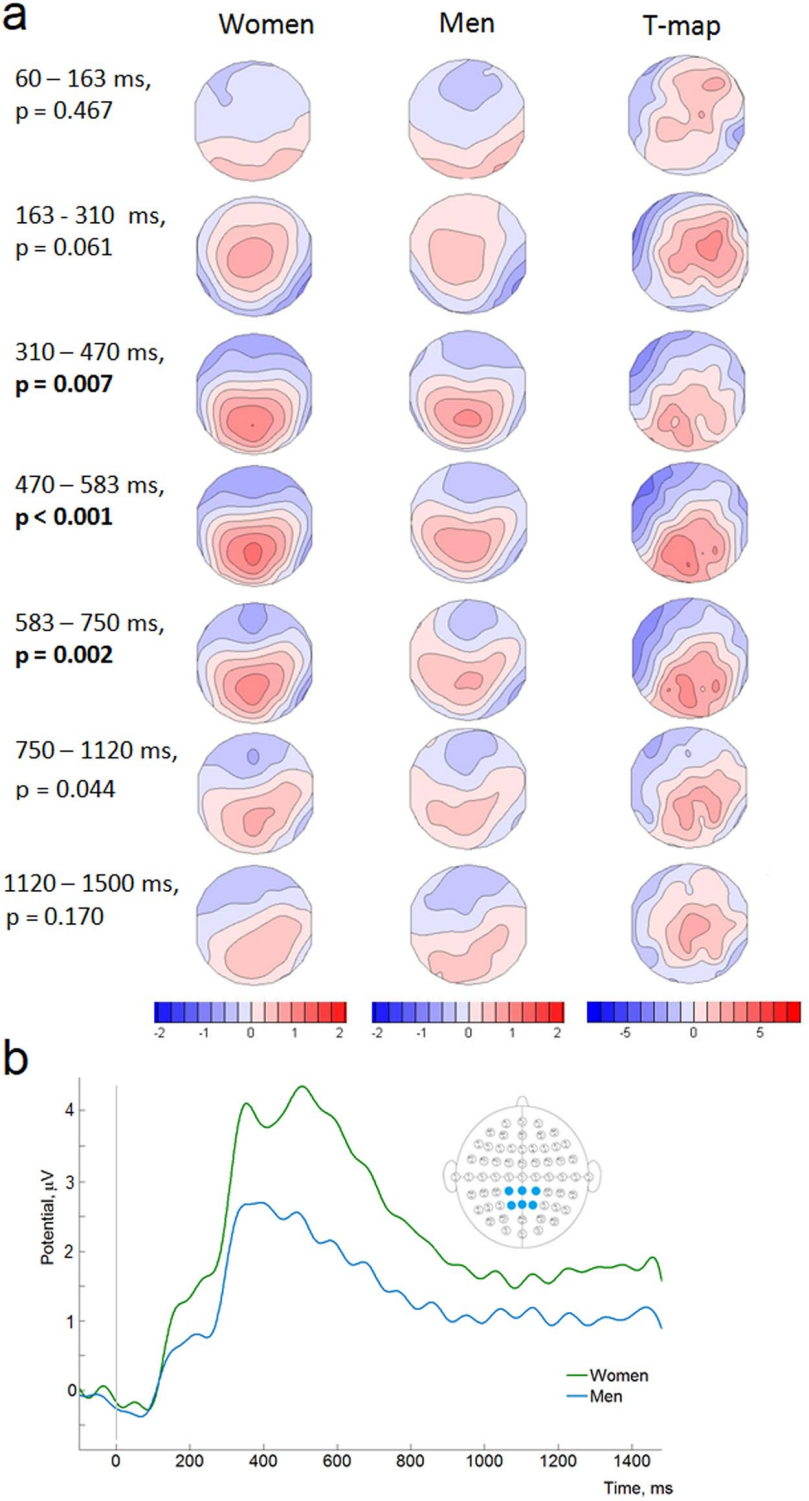
Topographic map and ERP waveform comparison between women and men. The p-values (randomization statistics) indicating significant differences are marked in bold. Blue map areas indicate negative, while red indicate positive values: (**a**) ERP scalp distributions and t-maps contrasting women minus men in 7 time periods after the stimulus onset averaged across all angular disparity conditions; (**b**) ERP waveform averaged over the parietal electrodes (CP1, CPz, CP2, P1, Pz, P2) in all angular disparity conditions in women and men.

TANOVAs demonstrated a significant effect of sex in the time interval from 310 to 750 ms (p ≤ 0.007, after the Bonferroni correction) (Fig. 6a). In this time window women displayed a left frontal negativity with the minimal t-value at the electrode FT7 (t = −4.49) as well as the bilateral parietal positivity with the maximal t-value at the electrode POz (t = 3.95) compared to men. Women also demonstrated a more positive ERP waveform over the parietal electrodes (Fig. 6b).

**Figure 7:**
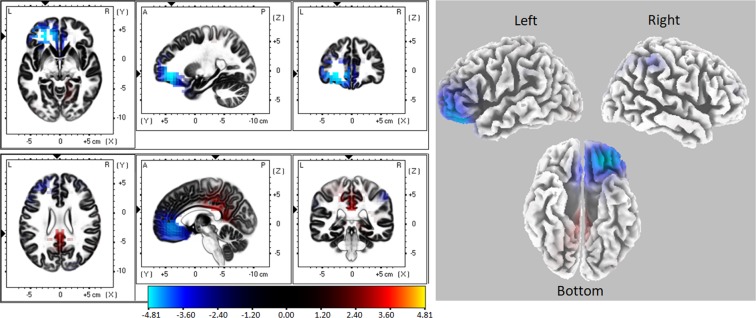
Source activity differences between women and men (women minus men) in the time window from 470 to 750 ms after stimulus (Object 2) onset. Areas marked blue indicate higher activation in men. The areas marked red and yellow indicate higher activation in women.

Source activity analysis (sLORETA) was performed in the time windows that demonstrated significant differences in topography (from 310 to 750 ms) and exhibited significant differences between women and men in the time window from 470 to 750 ms (Fig. 7 and Table 3). Men showed higher activation in the left frontal lobe (Middle Frontal Gyrus, Superior Frontal Gyrus, Anterior Cingulate, Orbital Gyrus, blue in Fig. 7), while women demonstrated increased activity in the Posterior Cingulate and Cingulate Gyrus (red in Fig. 7).

**Table 3:** Regions showing significant sex-related differences (women minus men) in the time window from 470 to 750 ms after stimulus onset.

X(MNI)	Y(MNI)	Z(MNI)	Voxel t-value	BA	Lobe	Structure
−25	40	−5	−4.81	11/47	Frontal	Middle Frontal Gyrus
−25	45	−15	−4.65	11	Frontal	Superior Frontal Gyrus
−15	45	0	−4.59	32	Limbic	Anterior Cingulate
−20	35	−25	−4.07	47	Frontal	Orbital Gyrus
−5	−35	25	3.17	23/31	Limbic	Posterior Cingulate
0	−35	25	3.16	23/31	Limbic	Cingulate Gyrus

Positive t-values indicate higher activation in women; the negative t-values indicate higher activation in men.

All regions were thresholded at p < 0.05 after the randomization procedure.

BA Brodmann’s area, MNI Montreal Neurological Institute.

### The effect of sex steroids

**Figure 8:**
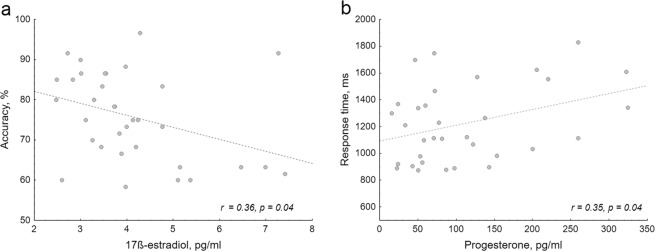
(**a**) The relationship between salivary 17ß-estradiol level and performance accuracy; (**b**) salivary progesterone level and response time in naturally cycling women. Reprinted from^92^, with permission from Elsevier.

We investigated the relationships between sex steroid (17-ß estradiol, progesterone and testosterone) levels and performance (ACC, RT, slope and intercept values) as well as the relationships between sex steroid levels and GFP (in the baseline condition and in the time window where sex had significant effect on GFP – from 320 to 990 ms) using Pearson correlations. First, we evaluated relationships that were previously described in the literature, such as: (i) the relationship between the performance accuracy and 17ß-estradiol level in women^21,22^; (ii) the relationship between the response time and progesterone level in women^10,23^; (iii) the relationship between performance accuracy and testosterone level separately for both sexes^22,24,25^. Additionally, we performed several exploratory analyses: (i) women RT *vs* 17ß-estradiol level; (ii) women ACC *vs* progesterone level; (iii) men and women slope and intercept values *vs* sex steroid levels; (iv) GFP values *vs* sex steroid levels. The main correlation analyses demonstrated a positive association between the response time and the levels of progesterone (r = 0.35, p = 0.046) as well as the negative relationship between accuracy and 17ß-estradiol level (r = −0.36, p = 0.040) in women (Fig. 8). However after corrections for multiple comparisons (each behavioural parameter was correlated with three hormones (0.05/3 = 0.017)), these correlations did not reach the level of significance, therefore should be treated as tendencies. There were no other significant relationships or tendencies between hormones and behavioural or ERP measures (all r < 0.29, all p > 0.23).

## Discussion

We used a modified 3D mental rotation task based on the Shepard and Metzler paradigm^54^ with a sequential object presentation to test sex-related differences in both behavioural and ERP measures. In line with previous studies, we have demonstrated several main findings, including: (i) a significant effect of angular disparity, which is indicative of the mental rotation process in the case of successive presentation of 3D figures; (ii) higher accuracy in men; (iii) significant differences in ERPs between men and women. We also found tendencies of a positive relationship between progesterone level and response time as well as a negative relationship between 17ß-estradiol and accuracy in women while testosterone did not show any significant associations or tendencies. The following discussion is thus structured focusing on the effects of angular disparity, sex, and sex steroids.

### Effect of angular disparity

Consistent with the previous findings^23,43,51,70,71^, the performance accuracy decreased while response time increased with increasing angular disparity. Slope and intercept values in our study were higher than demonstrated using successive paradigm with 2D shapes^30^. ERP analysis confirmed the previously demonstrated decrease in positivity over the parietal electrodes for higher angular disparity condition^32,39,43,48,51,70^. Together these findings indicate that a modified Shepard and Metzler paradigm with sequential object presentation evokes mental rotation characteristic responses in both behavioural and ERP levels. This result further justifies the usage of the modified Shepard and Metzler paradigm for the investigation of sex-related differences in mental rotation.

ERP differences between low and high angular disparity conditions first manifested as an increase in the general brain activation (higher GFP from approximately 270 to 460 ms in the high angular disparity condition) followed by the significant differences in topographies and source activation patterns (from approximately 470 ms). Such a time course roughly corresponds to the previously demonstrated sequence of the mental rotation sub-processes^32^. Accordingly, the initial time period before the emergence of GFP differences (before 270 ms) could be attributed to the sensory processing and simple evaluation^32,40,42,43^. The next time window (270–460 ms), characterized by an increased GFP in the high angular disparity condition, may be related to the evaluation of more complex aspects of the stimuli and strategy selection^32,44^. The higher GFP suggests a more challenging process^72,73^ for large angles. Finally, the last time period (from approximately 470 ms) can represent the rotation^32,39,40,43,47,48^ or the implementation of some other strategy to perform the task. The differences in topographies suggest the involvement of different brain sources depending on the difficulty of the task (high *vs* low angular disparity). In low angular disparity conditions more positive ERP values (seen from topographic point of view) and higher activation (seen from source localization) were concentrated mainly in the parietal area. Meanwhile, more widespread activation was evoked by the high angular disparity. In the time window from 470 to 583 ms, sources in frontal, temporal and occipital lobes and insula of the left hemisphere (sLORETA analysis) were significantly more activated in high, as compared to low, angular disparity condition leading to the assumption that the angular disparity-related separation of the strategies is occurring at approximately 470 ms after stimulus onset. This hypothesis is reinforced by the flattening response-time slopes at higher angular disparities, supporting a potential strategy switch, and echoing results from a previous mental rotation study^8^. Based on shallower slopes above 90 degrees, Boone and Hegarty^8^ suggested that the difference in RT slopes is related to the fact that mental rotation in the lower angular disparity trials is relatively easy and automatic, whereas for larger angles, when rotation is more demanding, it is more beneficial to switch to an alternative, e.g. orientation independent analytical strategy. Based on the slope change from steeper to flatter in our study, we also suggest a parallel switch from global to analytical strategy. This hypothesis is partially supported by the stronger activity in the left hemisphere for larger angles as the analytic strategy is more likely to involve the left hemisphere^70,74^. In addition, an alternative explanation of widespread activation of the left hemisphere evoked by high angular disparities, may be based on findings of studies, focusing on cerebral lateralization. For example Schintu *et al*.^75^ in their study with a large number of brain injured patients showed involvement of the temporal and frontal regions of the left hemisphere in a discrimination and recognition of more complex objects. Whereas visuospatial processing was mostly related to the posterior parietal cortex^75^. Based on this, we may speculate that higher angular disparity condition in our task required more complex processes during objects analysis and therefore evoked stronger activation in the frontal, temporal and other areas of the left hemisphere.

### Effect of sex

In concordance with other studies, men demonstrated significantly higher accuracy^5,10,12,76^ with the effect size (d = 0.51) comparable to that found using classical Shepard and Metzler paradigm (d = 0.48)^77^. Findings regarding the response time are consistent with studies reporting similar response times between sexes^32,41,43,51,78,79^. However, others demonstrated shorter response times in men^9^ as well as more subtle differences where men outperform only women with high levels of progesterone^10^, implying that sex hormones might modulate the performance. While in this study, we showed similar RT between men and women, we also showed tendency which replicates previously demonstrated increases in response time with increasing progesterone level in women^10,23^. The effects of sex steroids are subsequently discussed in the next section.

Higher general brain activation and higher relative positivity over the parietal scalp area in women during the rotation-related time period indicate greater cortical involvement. A positive relationship between GFP, ERP amplitudes and the effort to maintain cognitive performance during the prolonged period of a cognitively demanding task was recently demonstrated by Wang *et al*.^73^. To our knowledge, no EEG study so far has compared global brain activation during mental rotation between men and women. We suggest that mental rotation evokes higher brain activation^80,81^ in women during the mental rotation sub-processes that are related to pre-rotational setup (GFP peak at approximately 230 ms) and the rotation itself (GFP peak at approximately 380 ms)^32,40,43,45^. Higher positivity over parietal areas in women has already been demonstrated using two-^32,40^ and three- dimensional^50^ stimuli but these results were not found using classical Shepard and Metzler paradigm^51^. Lower ERP amplitudes over the parietal area were previously demonstrated in high-performers of a 2D mental rotation task suggesting more efficient brain functioning^39^. We therefore extend this interpretation to the results of our study and propose that men potentially performed mental rotation more efficiently as their accuracy was significantly higher, despite lower parietal ERP amplitudes and general brain activity. This finding is supported by the concept of neural efficiency, stating that individuals showing higher IQ and/or higher abilities in some specific task (e.g. spatial, verbal, etc.) display a lower brain activation while dealing successfully with a certain task (for review see^82^).

In addition to the lower overall brain activity and smaller ERP amplitude over the parietal area, men demonstrated higher activation in the left frontal cortex during the rotation-related time period (from approximately 470 ms). These results suggest that sex differences manifest not only through differential general brain activation, but may also depend on the activation of different brain areas. Such increases in left frontal activity in men, along with the higher accuracy, is in line with previously demonstrated findings where an increased activity in the left frontal cortex, together with motor and parietal cortices (contralateral to the performing hand), preceded only accurate task performances^83^. Regardless of the above-mentioned correspondence to some other studies, the finding of higher parietal activity in women and higher left frontal activity in men contradicts most of the fMRI studies showing the opposite result: higher levels of activation in the parietal region in males, but also higher activations in the frontal lobe in females^5,14,15,17–19^. The comparison of the results from the EEG and fMRI studies is complicated due to the inherent differences between the two methods. Although EEG has good temporal resolution, its spatial resolution is relatively poor, and vice versa for fMRI. Therefore, neuroimaging techniques are likely to provide a global image of sensory, perceptual, cognitive, and motor elements of a given mental-rotation task. In the present ERP study, we found a significant difference in the relatively short time-window from 470 to 750 ms. The results of a simultaneous EEG and fMRI study showed that left frontal and bilateral parietal areas are linked to the dynamical network during mental image transformation^36^.

Differential brain activation also supports the previously described assumption that men and women use different strategies to perform the task^84^. fMRI studies showed that activation of parietal areas is related to the transformation-specific computations, whereas activity in the frontal and especially the left frontal region is reflecting motor simulation during mental rotation^85^. The idea of different strategies, however, is not supported by our behavioural data, showing no differences in RT slopes between sexes. Moreover, a similar change of slope from steeper to flatter beyond 75 degrees is evident in both men and women and indicates a similar change of strategies in both groups.

### Relationships with sex steroids

In the present study we demonstrated tendencies which are in line with a well-established negative relationship between estradiol and performance accuracy (e.g.^5,21,22^) as well as a previously demonstrated increase in response time with increasing progesterone level^10,23^ in women. The effect of estradiol on spatial cognition could be explained through its relationships with the activity and volume of prefrontal, posterior parietal cortex and hippocampus, i.e. brain areas closely related to the spatial strategy, decision making, and transformation-specific computations^5,21,86^. Progesterone can influence cognition and behaviour through binding to the GABA receptors and exerting an inhibitory effect on a neural signal transmission (for review see^87,88^). In addition, communication between the two hemispheres, which has been shown to have high importance in mental rotation performance^36^, can also be affected by sex steroids. Estradiol can increase activation of both hemispheres (dominant and non-dominant for a given task) by activating the glutamatergic system, whereas progesterone may inhibit interhemispheric inhibition, thereby increasing activation in the non-dominant hemisphere for a given task via action on GABA receptors (for a review see^89^).

Although multiple studies reported the relationship between testosterone and mental rotation performance (e.g.^22,24,25^), we did not find any association. While this negative finding contradicted our hypothesis, several studies also failed to detect such a relationship^4,90^. In addition, it has been shown that the association between testosterone and mental rotation performance among men and women is nonlinear^23^, but our study sample might be too small to observe such interactions.

The exploratory analysis relating sex steroids and brain activity parameters did not yield any significant associations. Aiming to evaluate the effect of sex steroids on brain activity during mental rotation, we calculated correlations between sex steroids and GFP (in the baseline condition and in the time window where sex had significant effect on GFP – from 320 to 990 ms). Variations in GFP may be related to changes in general brain activity as well as to spatially specific activations, which, unfortunately, cannot be identified using ERP method. Whereas results of neuroimaging studies demonstrating significant effect of sex steroids on brain activity, suggest that correlations are sex-, hormone- and region-specific^5^.

### Limitations

The conclusions of the presented study may be limited by a quite small sample size, which possibly did not allow to detect smaller effects and possibly caused not significant correlations between behavioural parameters and sex steroids. In addition there are several methodological restrictions. First, both task performance and brain activity could be affected by several general traits, such as general intelligence and spatial abilities. These traits were not evaluated within the study design. Spatial abilities were evaluated using self-report, but the assessment of these abilities via a specific test would have been more reliable.

Second, solving a mental rotation task requires working memory, i.e. the ability to maintain an active representation of the object while simultaneously rotating the image^76^. In our study the encoding, retention and retrieval of the subsequently presented objects were essential for the correct task performance. The results of some previous studies suggests that working memory does not play the main role in sex differences demonstrated during mental rotation^48,76^. However, the interpretation of the results may benefit from an inclusion of working memory comparison between groups in future studies.

Third, the number of trials per one angular disparity condition was relatively low (n = 20). In addition, the number of valid trials decreased with increasing angular disparity due to the higher number of incorrect answers. Thus, we cannot exclude the possibility that some effects were unnoticed due to insufficient signal to noise ratio. The magnitude of this limitation is diminished by the statistical methods (randomization statistics) that were applied for whole scalp EEG analyses. Randomization statistics have a high statistical power even when an amount of data is comparatively small^59,91^. However, implementing more trials per single angular disparity condition and more angular disparity conditions would enhance the experimental design. Specifically, such improvements would allow analysing separate angular disparity conditions without excluding 25 and 100 degrees, and without aggregating conditions to low and high angular disparity categories. In addition, this would allow a better assessment of performance, as well as ERPs dynamics, and could increase the task sensitivity to an interaction between angular disparity and sex allowing the evaluation of the effect of angular disparity separately for men and women.

Forth, the levels of sex steroids can differ significantly between subjects. Testing women only once during a specific phase of the menstrual cycle, without an intra-individual hormone assessment (e.g. during two phases of the menstrual cycle), limits the interpretation of the effect that sex steroids exert on cognitive functions and brain activity.

## Conclusions

To summarize, we conclude that the modified Shepard and Metzler paradigm with the sequential stimuli presentation is suitable for studying mental rotation on both behavioural and ERPs levels. We demonstrated significant increase in response time, decrease in accuracy, and the decreased positivity of parietal ERP wave with increasing angular disparity between figures in a pair. Higher rotation angles were associated with an increased GFP during the time period possibly related to the evaluation of the complex stimuli aspects and strategy selection. This suggests an increased need for resources at larger angular disparities. Moreover, different RT slope values and differences in brain activation topographies suggest that separate strategies could have been used for different angular disparities.

We also demonstrated higher performance accuracy in men despite lower general brain activation and lower amplitude of parietal ERP wave, while there were no sex differences in response time. Performance accuracy in women tended to be negatively related to salivary 17β-estradiol level while the response time tended to increase with increasing progesterone. Although behavioural data did not support the hypothesis about different strategies used by men and women, differences in brain activation topographies suggests the involvement of different brain sources in both pre-rotational and rotation-related time windows. However, due to indirect evaluations we can only draw indirect conclusions concerning strategy use.

Overall, the work presented here provides some evidence that angular disparity and sex-related differences in mental rotation are complex and dynamic. Therefore, more studies implementing methods that allow for the evaluation of the precise temporal dynamics of this process would help to develop a much broader understanding of the mechanisms underlying such differences.

## Methods

This study is an extension to the paper published by our group^92^. The specifics of the mental rotation task, the demographic, hormonal, and behavioural data of mostly all participants is shared between the studies, therefore some descriptions in the Methods section overlap between these two papers^92^.

### Participants

72 healthy volunteers with no history of mental, neurological or hormonal disorders and with normal or corrected-to-normal vision participated in the study. Participants were recruited through advertisements at the university and on social networks. The data of eight participants were omitted from the further analyses due to the following reasons: (i) the average performance accuracy was lower than 50% (three women whose overall performance accuracy was lower than 50% were excluded seeking to avoid random answering; when accuracy rate is very low it could be that the observed sex differences may not be sex differences in mental rotation abilities per se, but may be due to the high number of errors^18^); (ii) irremovable EEG signal artefacts (one woman and two men); (iii) mean level of the Global Field Power more than 3 SD higher than average GFP level of the whole sample (a woman); (iv) extremely high level of sex hormones (a woman: the level of progesterone and the level of 17β-estradiol were more than 3 SD higher than mean group values). The data of 29 men and 34 women were used in the final analyses.

The final sample consisted of 32 university students and 31 working professionals. 8 women and 10 men were working or studying in the area related to law, finances, humanitarian, and social sciences; 26 women and 19 men were working or studying in the area related to biomedicine, physical sciences or technologies. As the literature has suggested that profession (e.g. arts *vs* sciences) may significantly affect mental rotation performance^93,94^, we investigated the possible effect of profession on mental rotation performance in our previous study and did not find a significant effect^92^. As we have used a sub-sample of our past study (naturally cycling women and men), we did not evaluate the effect of a profession in the current study.

There were no statistically significant differences between men and women with respect to age (men 24.5 ± 2.9; women 23.4 ± 3.0, t = 1.41, p = 0.16, d = 0.37) and years of education (men 16.2 ± 1.7; women 15.6 ± 2.0, t = 1.28, p = 0.20, d = 0.32).

Aiming to assess subjects’ self-perception about their spatial and mathematical abilities, we included the subjective evaluation of these abilities. Three categories: skills (how good are you in the field), hobbies (how much do you like to engage in the described activity) and experience (how experienced are you in the field), were used to describe aforementioned abilities. Questions were based on a psychometric Likert scale (from 1 to 5). Participants were asked to select a rating for their abilities in each category on a scale that ranged from ‘very weak’ (1) to ‘very strong’ (5). They were instructed to choose a number that best described their skills in the specified area. Examples were provided relating to each ability: spatial abilities (e.g. mental object manipulation in space, playing Tetris, 3D object design etc.), and mathematical abilities (e.g. solve a mathematical equation, brain teasers etc.). Mean values of every characteristic were obtained. There were no significant differences between men and women in self-assessed spatial (men 3.59 ± 0.73; women 3.21 ± 0.86, t = 1.83, p = 0.07, d = 0.48) and mathematical abilities (men 3.66 ± 0.81; women 3.36 ± 1.03, t = 1.23, p = 0.22, d = 0.32).

Only women with a regular menstrual cycle (ranging from 26 to 34 days) and not using hormonal contraceptives for at least three months were recruited for the study. Women participated in experiments either in the early follicular (from the 2^nd^ to 7^th^ day, n = 15) or mid luteal (from 16^th^ to 26^th^ day, n = 19) phase of their menstrual cycle as the reference points of low and high 17β-estradiol and progesterone levels respectively.

Bioethical approval was given by the Lithuanian Bioethics Committee. All participants signed the informed consent to participate in the study. The study was performed in accordance with the declaration of Helsinki.

### Mental rotation

We implemented the Shepard and Metzler paradigm with sequential figure presentation^54,95^: 3D figures (asymmetrical assembles of ten cubes on a black background from the ‘Library of Shepard and Metzler type Mental rotation stimuli’^53^) were presented in a sequential order (Fig. 1). Subjects were asked to evaluate whether the two figures were identical or the mirror images of each other, despite their angular disparity, as quickly and accurately as possible.

The task (Fig. 1) included 120 trials with 60 identical and 60 mirrored pairs. Figures were rotated around the vertical axis. Six angular disparity conditions (25°, 50°, 75°, 100°, 125°, and 150°) between figures in pair were used. Trials with different angular disparities were presented in randomized order for each participant. Participants were instructed to keep their gaze on the fixation point throughout the task and to respond with the dominant hand by pressing buttons of the PST Serial Response Box (PST, Inc.) – a green button for identical and a red for mirror figures. The fixation was not observed directly, but the saccades were recorded via electrodes applied to the temples. The retrospective evaluation of the electrooculogram signal proved that, in large, gaze was kept on the fixation point during the task. Feedback (a green cross for a correct and a red cross for an incorrect answer) followed each trial. The decision to provide feedback was made after the pilot experiments, where two experimental designs, with and without feedback, had been used. The participants reported that the information provided by the feedback helped them to perform at a higher level. Performance was evaluated by accuracy (ACC, %) and response time (RT, ms) for correct answers. Response time was defined as the time from the presentation of Object 2 to a button press. Response times shorter than 200 ms were excluded from analyses (two trials).

In addition, we computed a linear regression of RT on the angle of rotation to calculate slope and intercept. The slope of RT indicates the average change in milliseconds per additional degree of rotation. The slope indicates the cost, in terms of time, associated with rotating the target object one additional degree. In contrast, the intercept (the predicted RT for the 0 degree orientation, i.e. point where the regression line crosses y axis), reflects the time required to decide if two identical objects are the same or different and is a measure of all non-rotation related processes^24^.

E-Prime 2.0 software was used for stimuli presentation and behavioural data collection.

### EEG recording

The EEG was recorded using ASA lab hardware and software (ANTneuro, Netherlands). An electrode cap (Waweguard, ANT Neuro, Netherlands) containing 64 Ag-AgCl electrodes configured in equidistant scalp sites following the common 10–20 system was used. Additional electrodes were applied on the temples as well as above and below the left eye for the registration of horizontal eye movements and blinks. AFz was used as a ground electrode and averaged mastoid electrodes served as a recording reference. The impedances were kept below 25 kΩ. EEG and eye movements/blinks were recorded continuously (band pass from DC to ~138 Hz, a digitization rate of 512 Hz).

### Saliva sampling and hormonal analysis

The levels of 17ß-estradiol, progesterone and testosterone for women and testosterone for men were assessed from saliva samples. Subjects were asked to avoid eating, drinking (except water), smoking or brushing teeth for at least 30 min before the experiment. Prior to the saliva sampling procedure, participants were asked to rinse their mouth with cold water. A minimum of 1 ml of saliva was collected into a special polypropylene sampling device (IBL SaliCap, Germany). All specimens were stored at a temperature of −80 °C before being assayed. Specimens with blood traces (even a slightly reddish colour) were discarded from hormone assessment. The concentration of free 17β-estradiol, progesterone and testosterone was determined using a commercially available kit for the enzyme immunoassay from human saliva (IBL 17β-estradiol Saliva ELISA, IBL Progesterone Saliva ELISA, IBL Testosterone Saliva ELISA). Analytical sensitivity for 17β-estradiol was 0.30 pg/ml, for progesterone – 3.8 pg/ml and for testosterone – 2.0 pg/ml.

### Design and procedure

Each experimental session included general information questionnaires, saliva sampling, EEG electrode placement, practice of the mental rotation task and the main mental rotation task. The general information questionnaire contained questions about demographics (age, education duration, profession or field of education), general health (e.g. Do you have any endocrinal disorders? Is your vision normal? How are you feeling today (before the experiment)?), and female menstrual cycle (e.g. Is your menstrual cycle regular? Have you been using oral contraceptives during the last three months period?).

Before the EEG recording, subjects practiced until their accuracy reached 70–75%. All participants reached the 70% accuracy after a minimum of one and a maximum of three practicing sessions, i.e. after 11–33 trials. All experiments were performed in the afternoon. Testing took place in a soundproof, light-isolated chamber at a constant temperature (20–22 °C). Participants were seated in a comfortable armchair while stimuli were presented in the centre of an LCD monitor placed 80 cm from the subjects.

### Analysis of behavioural data

Data analysis was limited to the trials with identical objects and involved only correct responses. Taking data only from correctly solved tasks is a common practice of mental rotation studies (e.g.^9,31,46^). Previous studies demonstrated that the processing of mirrored objects differs from that of identical orientation^31,96–98^, therefore, in the present study, we decided to limit the analyses only to the trials with identical objects. On average it was 46.3 ± 7.8 identical and correctly solved trials per subject, 7.72 ± 1.03 trials per condition.

Accuracy (ACC, %) and response time (RT, ms) were analysed as the main behavioural variables. A 6 × 2 mixed design analysis of covariance (Mixed design ANCOVA) was used, including six angular disparities as a within-subject repeated measures factor, sex (male *vs* female) as a between-subject factor, and age, duration of education, spatial and math abilities as the covariates. Sphericity assumption for repeated measures was checked with the Mauchly’s Test of Sphericity. In cases where the assumption of sphericity had been violated, Greenhouse-Geisser results from corrected tests are reported. Two-tailed t-tests were used to compare slopes and intercepts between groups. Effect size was evaluated by partial eta squared (η^2^) or Cohen’s d (d) as appropriate.

A probability value p < 0.05 was taken as statistically significant. Statistical analyses were performed with the IBM SPSS Statistics software.

### ERP analyses

EEGLAB for MatLab©^99^, RAGU (Randomization Graphical User Interface)^60^ software was used for the ERP analysis and sLORETA^100^ (Standardized low-resolution brain electromagnetic tomography, http://www.uzh.ch/keyinst/loretaOldy.htm) software was used for the source localization.

The recorded EEG data were offline re-referenced against the average reference and digitally filtered in the 0.1–48 Hz range following the visual inspection where portions of the data containing coarse artefacts were removed. In addition, the Cleanline EEGLAB plug-in was used to remove the line noise. Artefactual electrode signals were removed and later interpolated resulting in the interpolation of 4.19% of all EEG electrodes. Visual inspection and manual artefact and artefactual electrodes rejection was based on the judgement of a person who was experienced in EEG and blind to the group and condition assignment. Independent Component Analysis (ICA) was applied to remove oculomotor and cardiac artefacts based on the waveform, the topography, and the time course of the ICA component^99^. ERPs were extracted from the pre-processed data by averaging single trials separately for participants, electrodes and experimental conditions (angular disparity) in the epochs starting −200 ms before and ending 1500 ms after the stimulus (Object 2) onset. Baseline correction was performed relative to a 200 ms pre-stimulus interval. Only identical object trials with correct answers were analysed.

#### Microstate model to define time periods for further ERP analyses

**Figure 9:**
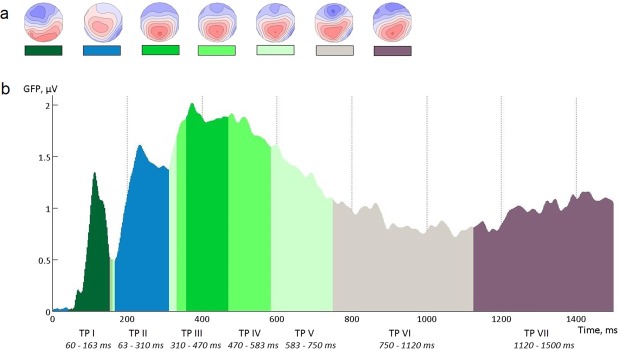
Microstate analysis of the mental rotation evoked ERPs for all subjects and all conditions. (**a**) Topographies of the clusters (blue map areas indicate negative, while red indicate positive values); (**b**) GFP values (vertical axis) as function of time (horizontal axis). Areas under the curve are coloured according to the topological maps in part a. TP – time period.

Time periods corresponding to separate sub-processes of a mental rotation were defined based on microstate analysis using RAGU^60^. Microstate analysis is one of the widely accepted tools for an assessment of functional state sequences in multichannel ERPs^101,102^. The idea of functional microstates is based on the hypothesis that each segment of a stable field topography reflects a temporally distinct functional entity, i.e. a certain information processing step^45,103,104^. The microstate model can be used to extract/define quasi-stable states or patterns of an EEG. To identify an optimal number of microstate maps, a cross-validation procedure was applied 50 times, testing between 3 and 15 microstates. The grand mean correlation in the test datasets initially increased with the growing number of microstates, before a plateau (r = 0.61) of 7 microstates classes was reached (for more details about microstate models selection and testing see^101,104^). Therefore, the 7-microstate model was chosen to extract the time periods for the further ERP analyses. That is, based on this microstate model, the time interval from 0 to 1500 ms after stimulus presentation was divided into seven time windows (Fig. 9).

#### ERP analyses and statistical evaluation

EEG data analyses and statistical comparisons were conducted using RAGU. RAGU compares scalp field differences between groups across all electrodes and epoch time points using rank order randomization statistics^59,60^. Five thousand randomization runs were conducted for each statistical test. The significance threshold was set to p < 0.05.

Global field power (GFP) calculations and topographic analyses of variance (TANOVA) were applied to evaluate the effect of the two main factors – sex and angular disparity, and the interaction between them.

We calculated GFP to asses quantitative (amplitude only) differences. GFP is a parametric assessment of a map’s strength which is independent of topography, computed as the standard deviation of the momentary potential values, and which shows the global strength of cortical activation^80,81^.

TANOVAs were used to determine qualitative differences between groups and experimental conditions. The TANOVA is an established method for comparing multichannel ERP data based on randomization techniques. It assesses the percentage of randomly shuffled data sets that show larger scalp field differences than the actual data, detecting topographic distribution of activity differences between groups and conditions. Significant TANOVA effects imply at least partially different sources of the ERPs (for more details about the TANOVA method see^60,61^). TANOVAs based on the amplitude-normalized (L2 normalization of the scalp field variance across sensors) maps were used, as possible amplitude differences were already evaluated by the GFP analysis. Normalization makes the TANOVA results independent of GFP and allows the separation of topographic effects from the different strengths of similar sources^59^.

Combining GFP and TANOVA, therefore, facilitates the data interpretation: GFP differences in the absence of topographic differences can be interpreted as a variation in the activity level of undistinguishable sources (brain areas), whereas a significant TANOVA effect indicates changes in the spatial distribution of activity, i.e. changes in the relative contribution of spatially separate sources.

TANOVA analyses were performed in the seven time windows selected based on the microstate analysis. This introduced the problem of multiple comparisons and so consequent Bonferroni correction reduced the level of significance to p = 0.007 (0.05/7) for the TANOVA results. For the GFP analysis randomization statistics in time-point by time-point mode was applied with level of significance p ≤ 0.05.

#### Source localization

The source estimation procedures were performed using the sLORETA software^100^. sLORETA is based on an inverse solution technique that standardizes a minimal norm inverse solution by source variance and measurement noise. The source activations were estimated using a realistic head model based on the Montreal Neurological Institute (MNI) 152 standard template. The source space was restricted to the cortical grey matter and hippocampal regions as determined by the corresponding digitized Probability Atlas, also available from the Brain Imaging Centre, Montreal Neurological Institute^100^. A total of 6238 voxels (5 × 5 × 5 mm resolution) were produced and source locations were given in the x, y, and z coordinate space (x from left to right; y from posterior to anterior; z from inferior to superior).

Source activity was computed and compared only for the time windows that showed significant between-group or between-condition differences in topographies since topographic differences assessed by a TANOVA must have resulted from differences in active brain regions. Statistical significance of the source differences was assessed by means of the sLORETA-built-in nonparametric randomization test which corrects for multiple comparisons^105^. The significance threshold was set to p < 0.05.

## Supplementary information

Appendix A
