# Supplementary material for: Mental rotation of sequentially presented 3D figures: sex and sex hormones related differences in behavioural and ERP measures
Source: Sci Rep. 2019 Dec 11;9:18843. doi: 10.1038/s41598-019-55433-y (PMC6906480; doi:10.1038/s41598-019-55433-y)
Supplement: Supplementary file 1 — Appendix A [file 41598_2019_55433_MOESM1_ESM.docx]

**Appendix A**

**Mental rotation of sequentially presented 3D figures: sex and sex hormones related differences in behavioural and ERP measures.**

*Ramune Griksiene^a*^, Aurina Arnatkeviciute^a^, Rasa Monciunskaite^a^, Thomas Koenig^b^, Osvaldas Ruksenas^a^*

^a^ Department of Neurobiology and Biophysics, Vilnius University, Vilnius, Lithuania.

^b^ Translational Research Center, University Hospital of Psychiatry, Bern, Switzerland.

^*^ Corresponding author. Ramune Griksiene: Department of Neurobiology and Biophysics, Vilnius University, Sauletekio ave. 7, 10257 Vilnius, Lithuania. E-mail: [ramune.griksiene@gf.vu.lt](mailto:ramune.griksiene@gf.vu.lt)


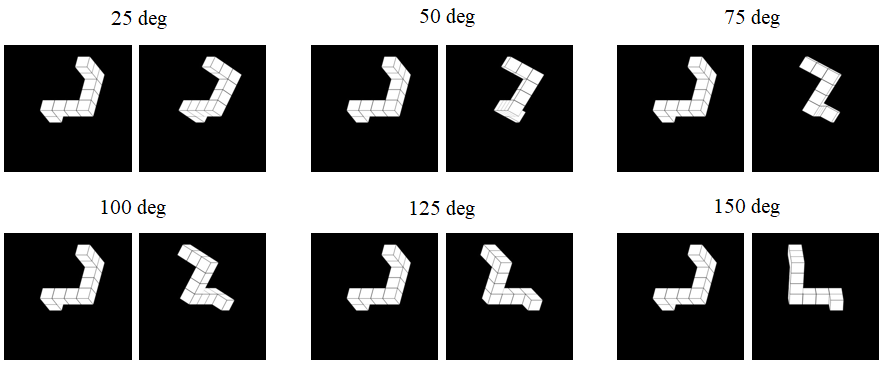


**Fig1** The examples of six angular disparity conditions.


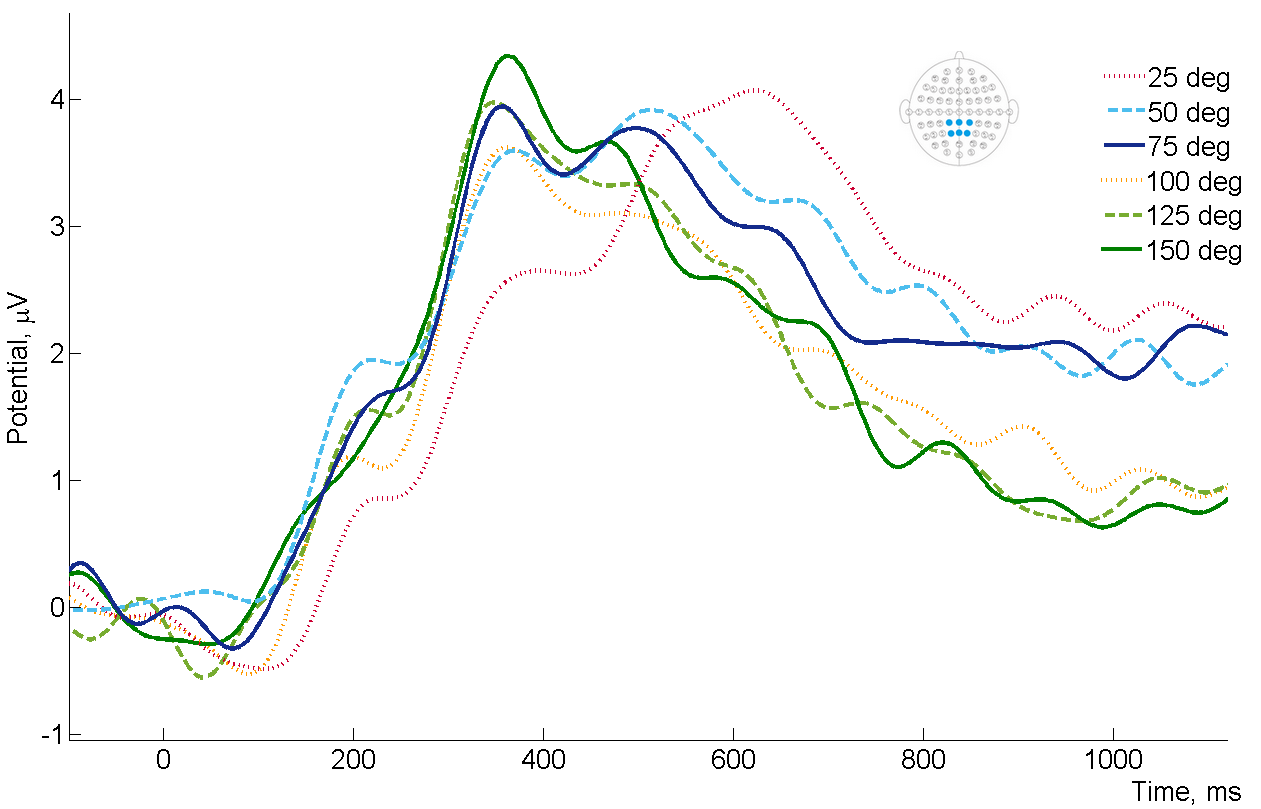


**Fig2** Averaged ERP waveforms from the parietal scalp area electrodes (CP1, CPz, CP2, P1, Pz, P2) of all subjects in six angular disparity conditions.
